# Novel Host Pathways Govern Epithelial Cell Invasion of Aspergillus fumigatus

**DOI:** 10.1128/spectrum.00084-23

**Published:** 2023-05-31

**Authors:** Hong Liu, Amol C. Shetty, Ashraf S. Ibrahim, Scott G. Filler, Vincent M. Bruno

**Affiliations:** a Division of Infectious Diseases, Lundquist Institute for Biomedical Innovation at Harbor-UCLA Medical Center, Torrance, California, USA; b Institute for Genome Sciences, University of Maryland School of Medicine, Baltimore, Maryland, USA; c David Geffen School of Medicine at UCLA, Torrance, California, USA; d Department of Microbiology and Immunology, University of Maryland School of Medicine, Baltimore, Maryland, USA; Universidade de Sao Paulo

**Keywords:** *Aspergillus fumigatus*, RNA-seq, airway epithelial cells, Af293, CEA10

## Abstract

Invasive aspergillosis is initiated when Aspergillus fumigatus adheres to and invades the pulmonary epithelial cells that line the airways and alveoli. To gain deeper insight into how pulmonary epithelial cells respond to A. fumigatus invasion, we used transcriptome sequencing (RNA-seq) to determine the transcriptional response of the A549 type II alveolar epithelial cell line to infection with strains CEA10 and Af293, two clinical isolates of A. fumigatus. Upstream regulator analysis of the data indicated that while both strains activated virtually identical host cell signaling pathways after 16 h of infection, only strain CEA10 activated these pathways after 6 h of infection. Many of the pathways that were predicted to be activated by A. fumigatus, including the tumor necrosis factor (TNF), interleukin-1α (IL-1α), IL-1β, IL-17A, Toll-like receptor 2 (TLR2), and TLR4 pathways, are known to be critical for the host defense against this fungus. We also found that the platelet-derived growth factor BB (PDGF BB) and progesterone receptor (PGR) pathways were activated by A. fumigatus. Using pharmacologic inhibitors, we determined that blocking the PDGF receptor or PGR inhibited the endocytosis of both strains of A. fumigatus in an additive manner. Both the PDGF BB and PGR pathways are also predicted to be activated by infection of A549 cells with other molds, such as Rhizopus delemar and Rhizopus oryzae. Thus, these pathways may represent a common response of pulmonary epithelial cells to mold infection.

**IMPORTANCE** Invasive aspergillosis is a deadly invasive fungal infection that initiates when Aspergillus fumigatus spores are inhaled and come into contact with the epithelial cells that line the airways and alveoli. Understanding this fungus-host interaction is important for the development of novel therapeutics. To gain a deeper understanding of how these airway epithelial cells respond to A. fumigatus during infection, we used RNA-seq to determine the transcriptional response of alveolar epithelial cells to infection with two different clinical isolates of A. fumigatus. Our analysis identified new host response pathways that have not previously been tied to infection with A. fumigatus. Pharmacological inhibition of two of these pathways inhibited the ability of A. fumigatus to invade airway epithelial cells. These two pathways are also predicted to be activated by infection with other filamentous fungi. Thus, these pathways may represent a common response of alveolar epithelial cells to mold infection.

## INTRODUCTION

Invasive aspergillosis, caused mainly by Aspergillus fumigatus, is a serious infection that afflicts patients who are immunocompromised due to cancer chemotherapy, organ transplantation, advanced HIV/AIDS, or high-dose corticosteroids (1, 2). Recently, it has been found that invasive aspergillosis is also a significant complication of patients with severe infections due to coronavirus disease 2019 (COVID-19) (3) and influenza A virus (4).

Invasive aspergillosis is initiated when airborne conidia are inhaled and then deposited into the airways and alveoli. These conidia are cleared by immunocompetent hosts. In susceptible patients, the conidia swell and then germinate to form hyphae that invade the epithelial cell lining of the airways. Because pulmonary epithelial cells are one of the first host cells to interact with A. fumigatus, there is intense interest in understanding how these cells respond to the fungus. Previous studies have found that A. fumigatus interacts with multiple pulmonary epithelial cell receptors, including dectin-1, integrin α5β1, ephrin type-A receptor 2 (EphA2), and E-cadherin (5–8). While each of these studies provide foundational knowledge of the mechanisms by which pulmonary epithelial cells respond to A. fumigatus, they are limited by their focus on single receptors.

To gain more comprehensive insight into the response of pulmonary epithelial cells to A. fumigatus, we and others have used either microarrays or transcriptome sequencing (RNA-seq) to analyze the transcriptional responses of the A549 type II pulmonary epithelial cell line to A. fumigatus (9–12). These studies have generally used Gene Ontology (GO) and KEGG pathway enrichment analysis to identify functional classes of host genes that are regulated in response to the fungus.

Previously, we analyzed the transcriptional response of two commonly used strains of A. fumigatus, Af293 and CEA10, to infection of A549 cells (12). Here, we determined the transcriptional response of A549 cells to these strains. We used the Upstream Regulator Analytic from the Ingenuity Pathway Analysis software (Ingenuity Systems) to identify signaling pathways that were potentially activated or repressed in A549 cells in response to infection with each of the A. fumigatus strains. We found that both strains of A. fumigatus activated the platelet-derived growth factor BB (PDGF BB) and progesterone (PGR) pathways. Pharmacologic inhibition of these pathways reduced the endocytosis of A. fumigatus by A549 cells in an additive manner. Also, we found that approximately one third of the signaling pathways that were upregulated in response to infection with A. fumigatus were also upregulated by infection with *Mucorales* species, which cause mucormycosis, suggesting that different mold infections activate a conserved set of signaling pathways in pulmonary epithelial cells.

## RESULTS

### RNA-seq analysis of *in vitro* Aspergillus fumigatus infections.

We performed RNA-seq analysis on poly(A)-enriched RNA isolated from monolayers of the A549 human type II pneumocyte cell line infected with A. fumigatus for 6 or 16 h. The A549 cell cultures were infected individually with conidia derived from two different well-characterized clinical isolates, Af293 and CEA10, thus allowing the identification of both common and strain-specific responses. Following addition to host cells, the resting conidia first swell and then begin to germinate. This is followed by rapid hyphal cell elongation and branching. Under our experimental conditions (see Materials and Methods), the A. fumigatus cells were present as a mixture of swollen conidia and early-stage germlings at 6 h postinfection. By 16 h postinfection, the entire host cell monolayer was covered by a hyphal mat (12). We observed no significant difference between the germination rates of Af293 and CEA10 under these conditions. Our analysis also included time-matched uninfected samples that served as negative controls. Each of the 6 experimental conditions (Table S1 in the supplemental material) was tested in triplicate. Because both fungi and humans are eukaryotes that produce polyadenylated messages, the RNA preparations contained a mixture of mRNAs expressed by A. fumigatus and the host cells. Our analysis of the A. fumigatus transcriptome in response to A549 cells, from the same sample set, is described elsewhere (12). From each of the 18 sequencing libraries, we obtained an average of 61.3 ± 24.7 million reads (mean ± standard deviation [SD]) that mapped to the human reference genome (Ensembl GRCh38) (Table S1). Analysis of human gene expression revealed 7,056 genes that were differentially expressed (false-discovery rate [FDR] of <0.01 and absolute log_2_ fold change of ≥1.0) during at least one infection time point, in response to at least one of the strains, compared to their expression in the appropriate uninfected control group (Table S2).

**FIG 1:**
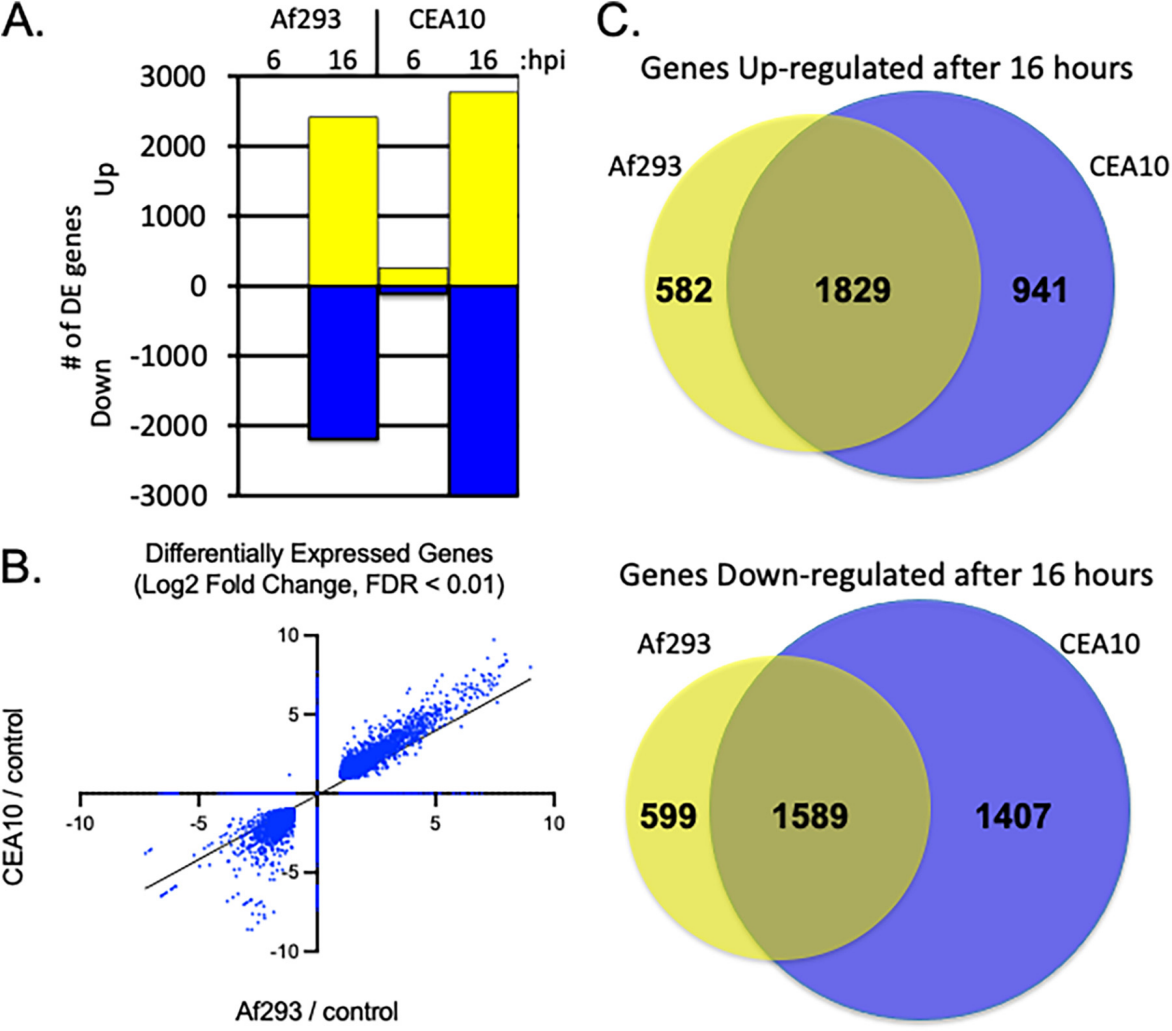
Host response to A. fumigatus infection. (A) The numbers of genes that were induced (yellow) or repressed (blue) by infection with each of the A. fumigatus strains at 6 or 16 h after the addition of conidia. (B) Scatterplot of differential expression data in response to 16 h of infection with each A. fumigatus isolate. Each point represents a different host gene. (C) Venn diagrams representing the overlap in the numbers of genes regulated in the same direction between infections with the two different isolates.

After 6 h of infection, the two strains of A. fumigatus induced significantly different epithelial cell responses. At this time point, infection with Af293 induced the expression of only 6 genes, while infection with CEA10 caused the differential expression of 366 genes (Fig. 1A). All six of the genes that were induced by Af293 were also induced by CEA10. These genes were *FOSB*, *FOS*, *ATF3*, *EGR1*, and *NR4A3* (Table S2). Interestingly, *NR4A3* specifies a transcriptional transactivator and the other five genes encode transcription factors. Thus, the activation of these transcription factor genes likely represents a core early response to A. fumigatus infection. As the infection progresses, the products of these genes likely induce many of the subsequent pulmonary epithelial cell responses.

After 16 h of infection, the transcriptional responses to each isolate were relatively similar. Although CEA10 induced differential expression of a greater number of genes than Af293 (Fig. 1A), the common set of differentially expressed genes were regulated to a similar extent (Fig. 1B). For each strain, the number of genes that were significantly upregulated was similar to the number of genes that were significantly downregulated. The sets of genes that were differentially expressed in response to each isolate were somewhat different (Fig. 1C). Given the extent of genomic diversity among A. fumigatus isolates and the lack of phenotypic data on a wide variety of isolates regarding host-pathogen interactions, we are reluctant to draw any conclusions about strain-specific responses elicited in A549 cells by the isolates examined here. The complete set of genes differentially expressed in response to each strain can be found in Table S2.

### Upstream regulator analysis of host gene expression.

**FIG 2:**
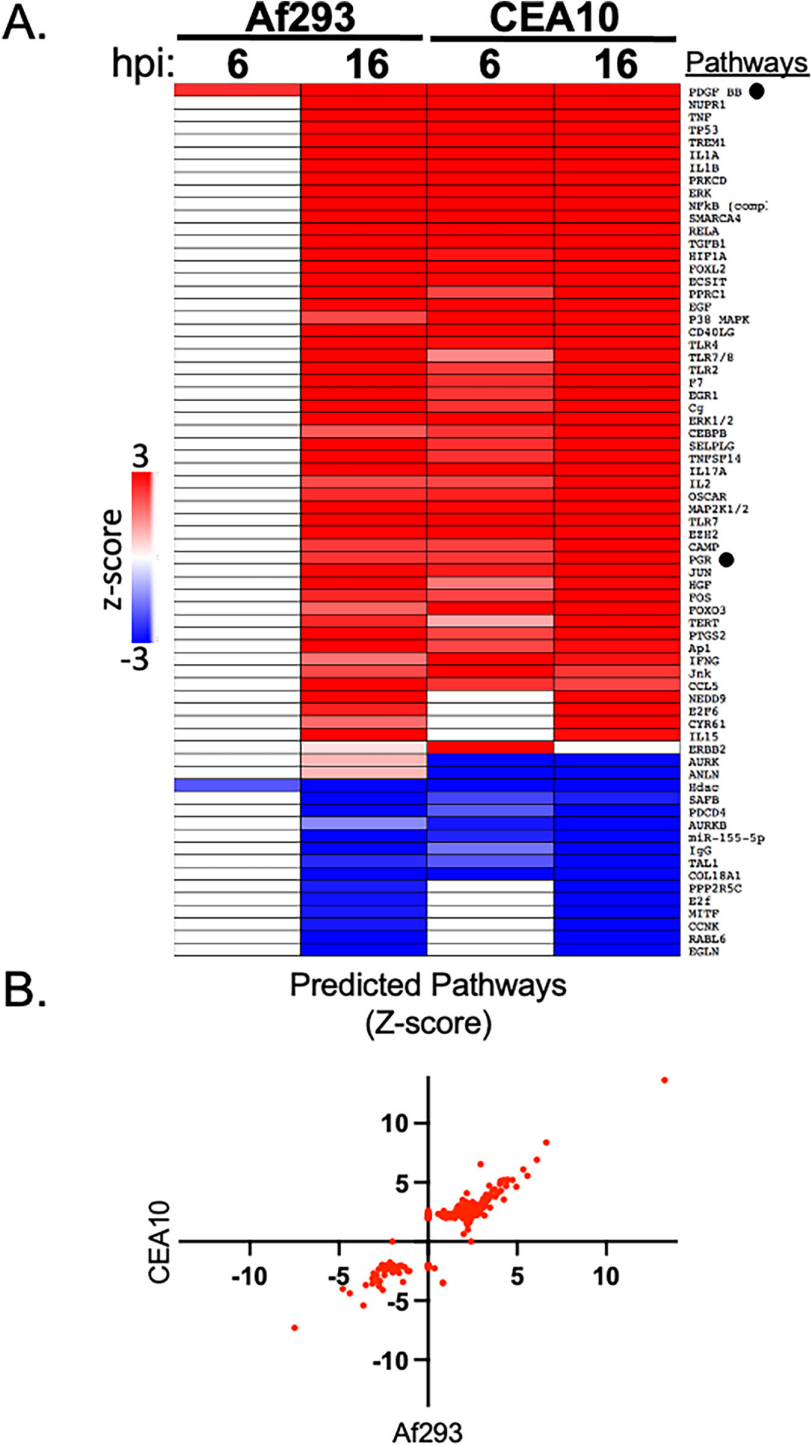
Upstream regulator analysis of A. fumigatus-induced gene expression. (A) Each regulator was predicted by Ingenuity Pathway Analysis (IPA) to be activated (red, z-score of >2) or repressed (blue, z-score of <2) during infection of A549 cells with either A. fumigatus strain Af293 or CEA10. Each of the depicted pathways achieved a z-score of ≥|3.0| under at least one of the conditions. White indicates no predicted activation or repression. Black circles indicate regulators of biological interest that were selected for follow-up experiments. (B) Scatterplot of z-scores for pathways modulated during at least one of the 16-h infections. Each point represents a different pathway as designated by the Upstream Regulator Analytic.

Network analysis of pathogen-responsive gene expression can be used to identify host signal transduction pathways that are relevant to the host-microbe interaction (13–16). We used the Upstream Regulator Analytic from the Ingenuity Pathway Analysis software (Ingenuity Systems; http://www.ingenuity.com) to identify signaling pathways that were potentially activated or repressed in A549 cells in response to infection with each of the A. fumigatus strains. Our primary focus was on the identification of pathways that behaved similarly in response to infection by each strain, as these pathways represent the core response of A549 cells to A. fumigatus. This analysis predicted the conserved activation (z-score of ≥2.0) of 113 different signaling pathways and the conserved repression (z-score of ≤−2.0) of 34 different signaling proteins (Fig. 2, Table S3). A pathway regulation was considered “conserved” if it met these criteria in at least one time point for each strain, allowing for differences in temporal dynamics of pathway regulation. Examination of the pathway modulation following 16 h of infection revealed that the majority of conserved pathways were activated, or repressed, to a similar extent by each of the A. fumigatus strains (Fig. 2B). Our network-based approach was verified by our identification of multiple host signaling pathways whose members are known to be upregulated or to play a role (based on genetic or inhibitor studies) in the host response to A. fumigatus. These pathways were activated in response to both A. fumigatus strains and included the TNF (tumor necrosis factor), IL1B (interleukin-1β [IL-1β]), NFKB (nuclear factor kappa B), IFNG (interferon gamma), ERK (extracellular signal-regulated kinase), p38 MAPK (mitogen-activated protein kinase), TLR2 (Toll-like receptor 2), TLR3, TLR4, IL-6, and AP-1 (activator protein 1) pathways (17–32).

Our analysis also predicted the activation of several pathways that have not been previously reported to play a role in the host response to A. fumigatus infection. These pathways include the PDGF BB (platelet-derived growth factor BB), PGR (progesterone receptor), NUPR1 (nuclear protein, transcriptional regulator 1), NEDD9 (neural precursor cell expressed, developmentally downregulated 9), FOXL2 (forkhead box L2), FOXO3 (forkhead box O3), and EZH2 (enhancer of zeste 2) pathways. The complete list of regulated pathways during each infection is presented in Table S3. We chose two pathways with predicted activation in response to both A. fumigatus strains, PDGF BB and PGR, for functional follow-up. Because both pathways showed predicted activation in response to each of the A. fumigatus strains, they are less likely to be part of a strain-specific host response.

### PDGF receptor (PDGFR) and PGR independently govern uptake of A. fumigatus into airway epithelial cells.

**FIG 3:**
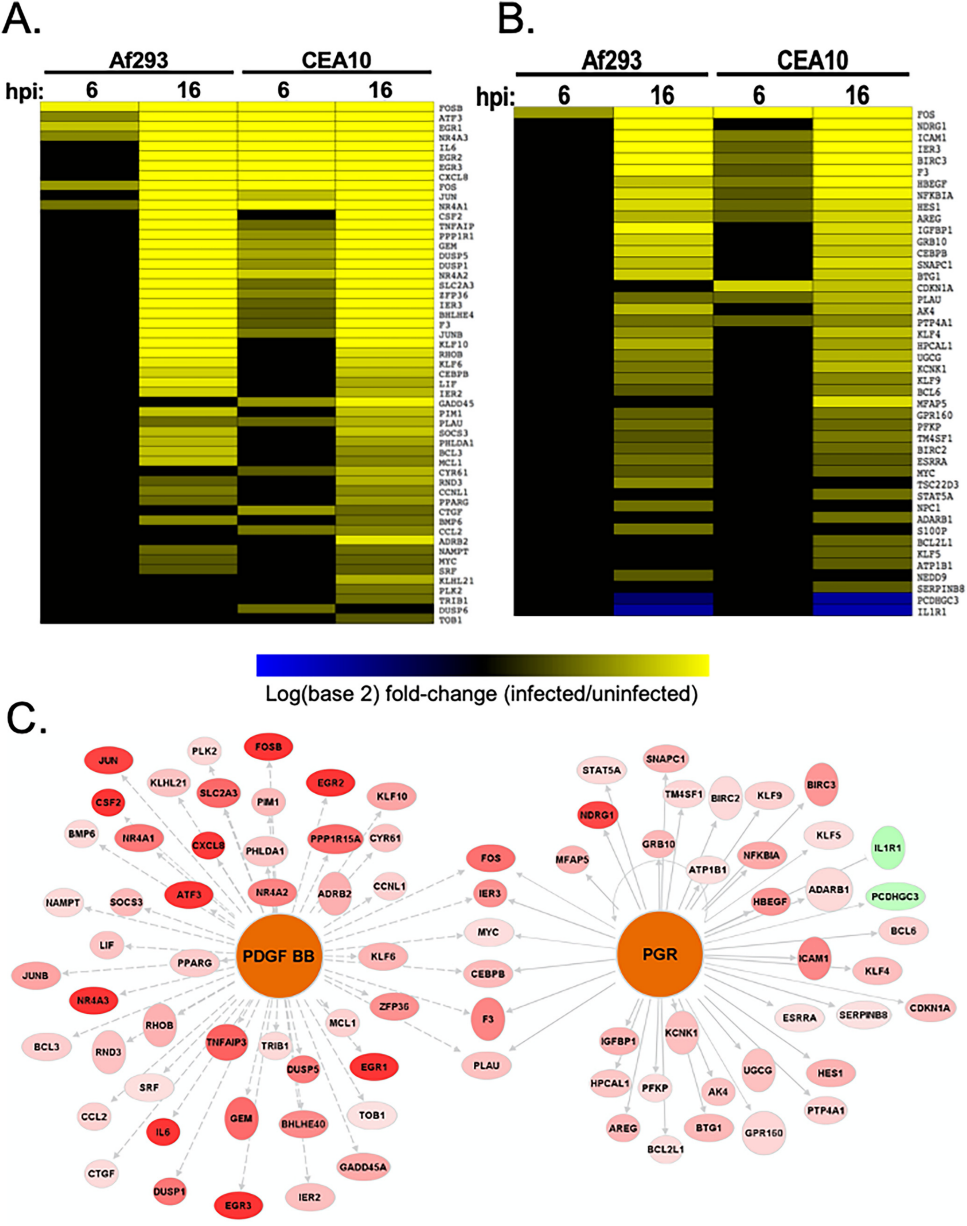
Regulation of PDGF BB and PGR pathways during *in vitro* infection. Differential expression of downstream targets of the PDGF BB pathway (A) and PGR pathway (B) during infection with each of the A. fumigatus strains at each time point is identified. Yellow indicates increased expression during infection; blue indicates reduced expression during infection; black indicates no change in expression during infection. (C) Network depiction of genes regulated by the PDGF BB and PGR pathways 16 h postinfection with the CEA10 strain. Red indicates genes that were upregulated during infection; green indicates downregulated genes.

Targets of the PDGF BB pathway were significantly regulated at both 6 and 16 h in response to both strains, while the downstream transcriptional targets of PGR were regulated at both 6 h and 16 h in response to CEA10 but only at 16 h postinfection in response to Af293 (Fig. 3A and B). Neither of these pathways has been previously reported to play a role in the host response to A. fumigatus. Among the 52 known PDGF BB transcriptional targets and 40 known PGR transcriptional targets that were differentially expressed following 16 h of infection, only 6 (11.5% and 15%, respectively) are known targets of both pathways (Fig. 3C), suggesting that PDGF BB and PGR mediate the regulation of distinct transcriptional modules in response to A. fumigatus infection (Fig. 3C).

**FIG 4:**
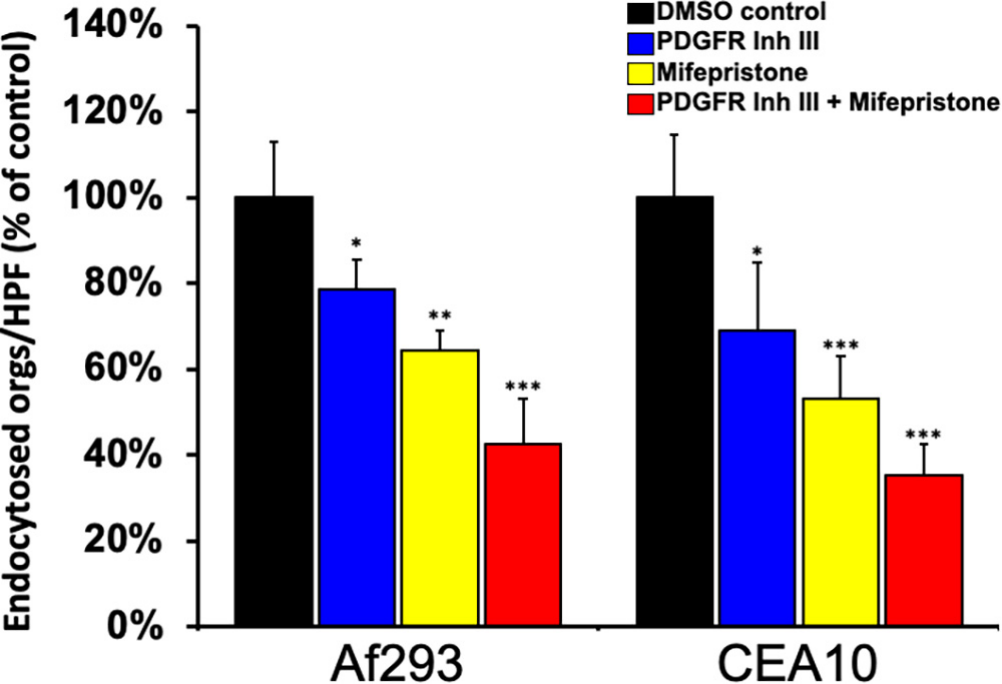
PDGF BB and PGR mediate endocytosis of A. fumigatus by airway epithelial cells. The effect of pharmacological inhibition of PDGF receptor, progesterone receptor, or both on induced endocytosis was examined. Results are the mean values ± SD from three experiments, each performed in triplicate. *, *P* < 0.01; **, *P* < 0.001; ***, *P* < 0.0001 compared to DMSO control by two-tailed Student’s *t* test assuming unequal variances. Orgs, organisms; HPF, high-power field; Inh, inhibitor.

The PDGF BB pathway was of particular interest because of the angioinvasive nature of invasive aspergillosis. Platelet-derived growth factors (PDGFs) are serum proteins with documented roles in angiogenesis, atherosclerosis, and cancer (33–37). There are four different PDGFs: PDGFA, PDGFB, PDGFC, and PDGFD. Each protein functions as a part of a secreted homodimer or heterodimer that binds to and induces the tyrosine phosphorylation of the PDGFR α and/or β subunits (33). Because PDGF BB homodimers can serve as ligands for the PDGFR α/α homodimers, β/β homodimers, or α/β heterodimers (38), these results suggest that A. fumigatus induces the phosphorylation of PDGFR and subsequent activation of cellular signaling through either the PDGFRα or PDGFRβ subunit. PDGFR signaling has been shown to facilitate invasion of endothelial cells and oral epithelial cells by Candida albicans and damage to endothelial cells by Rhizopus delemar (14, 16). Both of these fungi, like A. fumigatus, enter cells by induced endocytosis (39, 40). We tested whether blocking PDGFR signaling would also protect alveolar epithelial cells from invasion by A. fumigatus by measuring the effect of a small-molecule PDGFR tyrosine kinase inhibitor on A. fumigatus-mediated endocytosis of A549 cells. Treatment of cells with PDGFR tyrosine kinase inhibitor III (CAS 205254-94-0) significantly reduced the endocytosis of germlings of both A. fumigatus strains (Fig. 4).

Progesterone (PG) modulates a variety of physiological processes, including pregnancy, lung development, sperm function, nervous system function, glucose tolerance, pancreas function, and breast cancer etiology (41–44). There are both cytosolic and membrane PG receptors. The cytosolic form of PGR exists as two well-characterized main isoforms, PR-A and PR-B, which are encoded by a single gene but transcribed from two different promoters (41, 45). Upon binding of progesterone, cytosolic PGR dimerizes, enters the nucleus, and binds to DNA at progesterone response elements (PRE) to activate progesterone-sensitive-gene expression (46). PG receptor membrane component 1 (PGRMC1) and PGRMC2 are expressed on the plasma membrane and function in a complex with other receptors, including epidermal growth factor receptor (47, 48).

We tested the relevance of PGR activation to infection by pretreating airway epithelial cells with an inhibitor of PGR activation, mifepristone. Mifepristone is structurally related to steroids, and it binds strongly to PGR and acts as an antagonist of classical nuclear PGR signaling (41, 49). However, it can act as either a progesterone agonist or antagonist for membrane PGR signaling, depending on the cell type (50, 51). We found that treatment of cells with mifepristone significantly reduced the endocytosis of both A. fumigatus strains (Fig. 4), to a slightly greater extent than treatment with the PDGFR inhibitor. Pretreatment of cells with both inhibitors resulted in a greater inhibition of endocytosis than pretreatment with each of the inhibitors alone, and the same effect was observed for both A. fumigatus strains (Fig. 4). Our pathway analysis and inhibitor studies, taken together, suggest that PDGF receptors and PGR function in separate pathways to govern endocytosis of airway epithelial cells by A. fumigatus.

### Correlates of A. fumigatus gene expression with host pathway activation.

The most striking difference in the host response that we observed between the two infections occurred at the 6-h time point, when strain Af293 infection induced the expression of 6 genes, while strain CEA10 infection induced the expression of 258 genes and the repression of 108 genes (Table S2). This remarkable difference was also evident in our pathway analysis (Fig. 2). We reasoned that the differential response at the 6-h time point could occur because the A549 cells were exposed to different sets of proteins expressed by the two A. fumigatus strains. To explore this possibility, we revisited our transcriptome analysis of both fungal strains during coincubation with A549 cells for 6 or 16 h (12), an analysis that was performed on the same infection samples presented here. Specifically, we looked for conserved A. fumigatus genes that were differentially expressed (FDR < 0.01) between the 2 strains at the 6-h postinfection time point.

**FIG 5:**
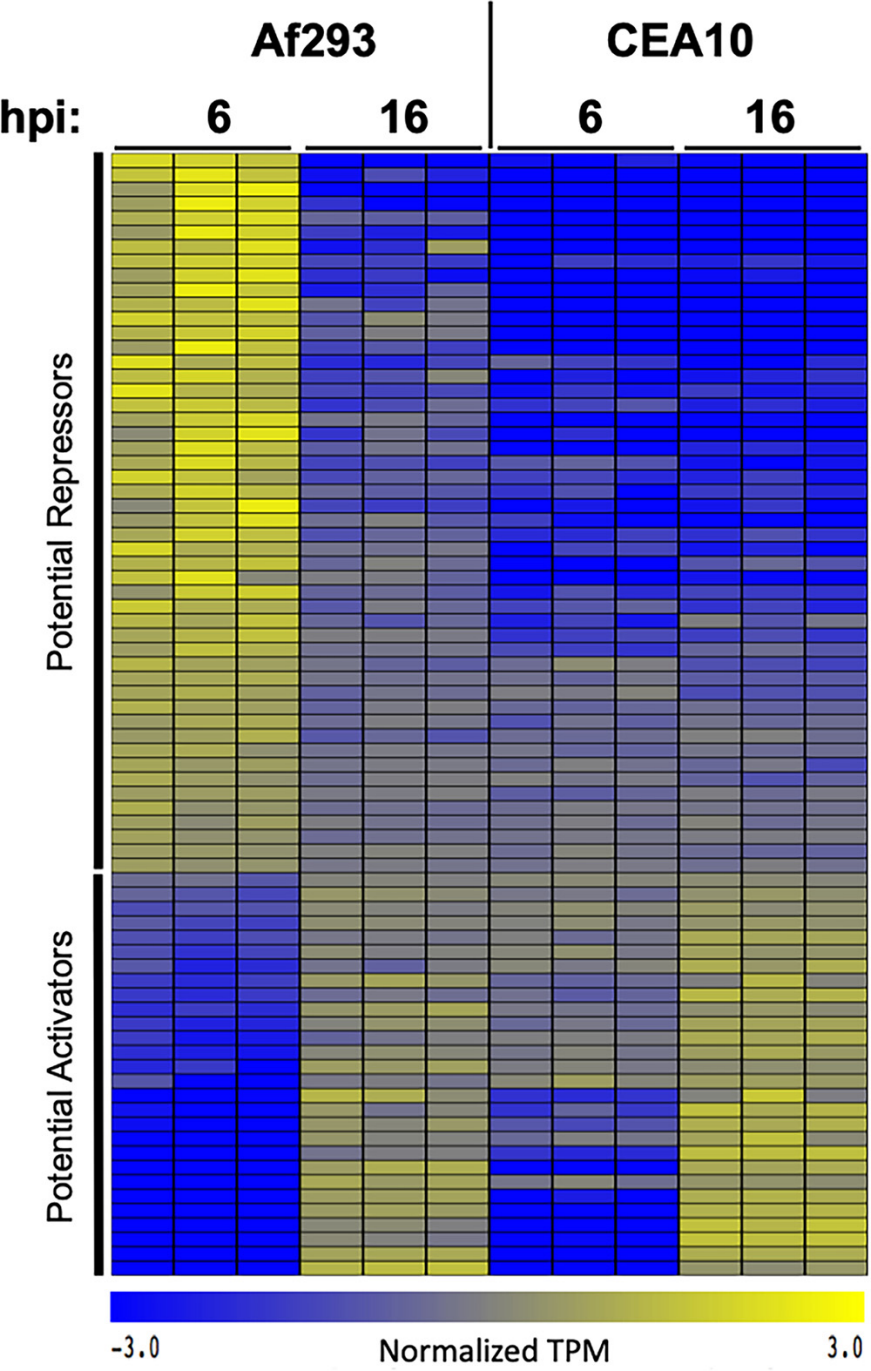
Correlation of fungal and host gene expression. A set of 78 A. fumigatus genes whose expression patterns correlated with the magnitude of host gene expression across all 4 infection groups. Each column represents an individual sample. Values represent log-transformed absolute expression (transcripts per million [TPM]) normalized across all samples.

We identified 148 genes that were more highly expressed in strain CEA10 and 413 genes that were more highly expressed in Af293 under these conditions. From this set of 561 fungal genes, we searched for genes whose expression patterns across all 12 samples (both A. fumigatus strains and both time points) correlated, either directly or inversely, with the overall trends in host gene expression observed in the same samples. The A. fumigatus genes with the highest expression in strain Af293 at 6 h postinfection represent genes that potentially inhibit epithelial cell transcriptional responses. Likewise, genes with the lowest expression in strain Af293 at 6 h postinfection potentially activate the epithelial cell response. We identified 78 genes with an expression pattern that matched, or was the inverse of, the overall host transcriptional response (Fig. 5, Table S4). Among these 78 genes, the expression of 28 appear to activate the host transcriptional response, while 50 of them likely repress the host response. Eight of these genes are predicted to have a secretion signal, and 12 are predicted to be localized to the cell wall or to an extracellular region (Table S4). This subset of genes is of particular interest because their potential cell wall or extracellular localization makes them more likely to be directly involved in the interaction between A. fumigatus and airway epithelial cells. Taken together, the set of genes with correlated and anticorrelated mRNA expression provides a rational set of testable hypotheses to explain the differences in host responses that are observed between different isolates of A. fumigatus.

### Common host responses to A. fumigatus and *Mucorales*.

We next wondered if the host transcriptional responses that we observed are specific to infection with A. fumigatus or are also part of the response of airway epithelial cells to filamentous fungi. This question is of particular importance given that both A. fumigatus and *Mucorales* species are angioinvasive fungi that cause invasive pulmonary infections in immunocompromised hosts. To begin to address this question, we used the Upstream Regulator Analytic to evaluate our data from a previous study in which we performed RNA-seq analysis of the response of A549 epithelial cells to 6 and 16 h of infection with R. delemar and Rhizopus oryzae, two species of filamentous fungi from the order *Mucorales* (14). We also analyzed RNA-seq data of A549 cells infected for 12 h with influenza B virus (52) and for 1 h with Pseudomonas aeruginosa (53).

**FIG 6:**
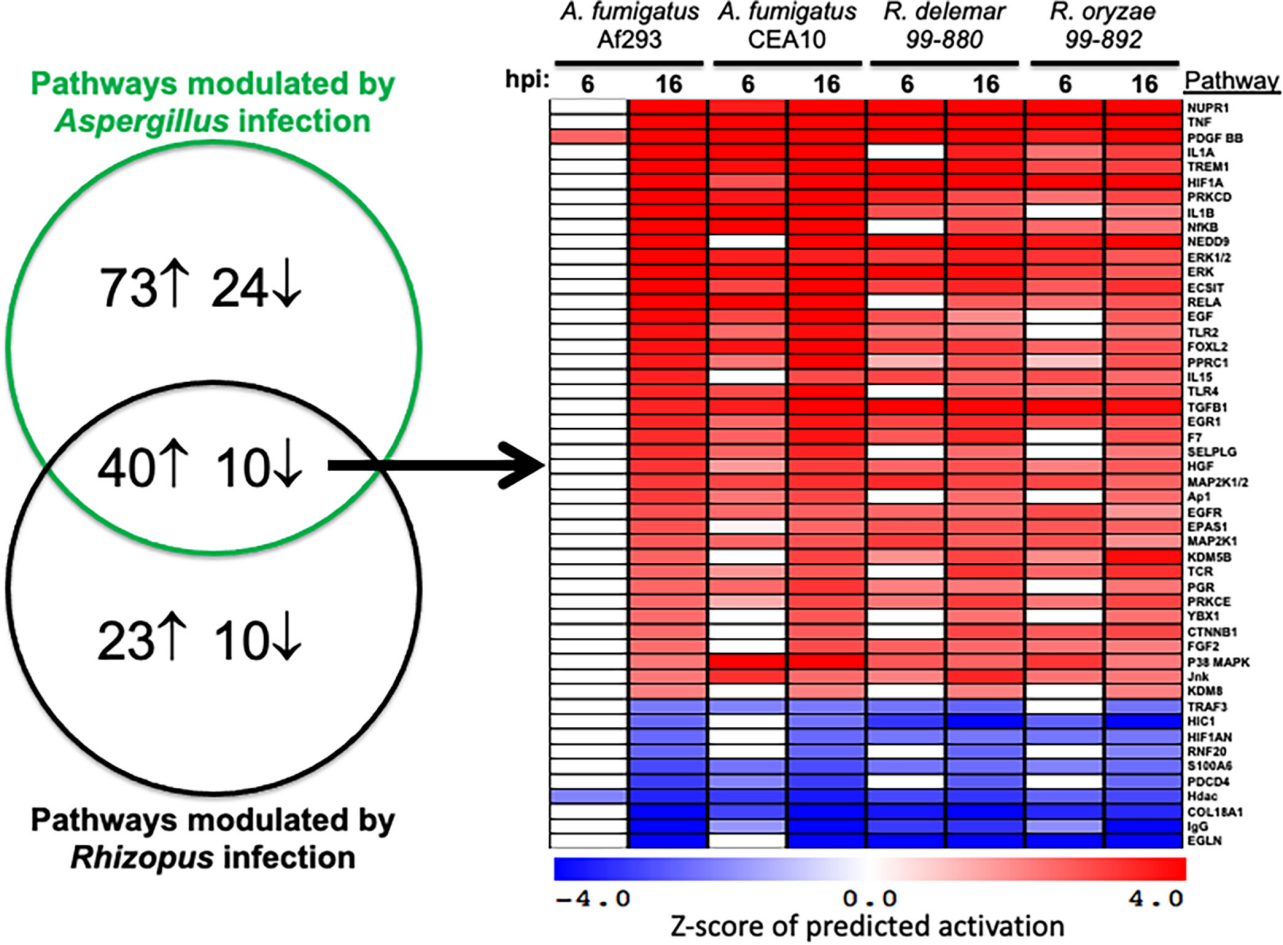
Defining common host responses to *in vitro* infection with filamentous fungi. (a) Overlap in pathways regulated during infection of A549 cells with A. fumigatus, R. oryzae, or R. delemar. (b) Upstream regulator analysis of host pathways. As in Fig. 2, red indicates prediction of an activated pathway (z-score of >2), blue indicates prediction of a repressed pathway (z-score of <−2), and white indicates no predicted activation or repression during infection of A549 cells with either A. fumigatus strain Af293 or CEA10.

Direct comparison of the upstream regulator analyses revealed that 50 of 147 host pathways predicted to be modulated in response to *in vitro* A. fumigatus infection are also modulated in the same direction following *in vitro* infection with the *Mucorales* species, including the two pathways studied here, PGR and PDGF BB (Fig. 6). The complete comparison of the pathway analyses in response to both A. fumigatus isolates and both Rhizopus isolates is presented in Table S5. The PDGF BB pathway, but not the PGR pathway, is also predicted to be modulated in response to influenza B virus and P. aeruginosa (data not shown). It should be noted that the P. aeruginosa response data were obtained at a single, early time point (1 h postinfection). Different results may be obtained if later time points are examined. Nevertheless, these findings suggest that activation of the PDGF BB pathway may be part of a common response of pulmonary epithelial cells to mold, bacterial, and viral infections. While activation of the PGR pathway may be more specific to mold infections, testing the response of A549 cells to additional pulmonary pathogens is required to verify this possibility.

## DISCUSSION

This study, in combination with that of Watkins et al. (12), represents a comprehensive dual-species analysis of A. fumigatus infection of airway epithelial cells *in vitro*. Using the Upstream Regulator Analytic, we found that two strains of A. fumigatus were predicted to activate a common set of signaling pathways. Approximately one third of these pathways are also modulated by infection of A549 cells with R. delemar and Rhizopus oryzae, highlighting significant commonalities in the transcriptional responses of pulmonary epithelial cells to these two genera of filamentous fungi. Previously, we found that Candida albicans infection of oral epithelial cells *in vitro* also induces transcriptional activation of the PDGF BB, NUPR1, NEDD9, FOXL2, TNF, IL1B, NFKB, ERK, and p38 pathways (16). Thus, these pathways likely represent a common epithelial cell response to fungal infection. Although there are limited data on the transcriptional responses of A549 cells to other microbial pathogens, the PDGF BB pathway appears to be activated by infection with influenza B virus and P. aeruginosa. Further studies are necessary to determine whether activation of the PDGF BB pathway represents a sentinel response of pulmonary epithelial cells to infection.

Although we were able to find only a few studies that analyzed the transcriptional response of A549 cells to bacterial or viral pathogens, we identified previous studies that used either microarrays or RNA-seq to determine the transcriptional response of A549 cells to A. fumigatus infection (9, 11). Using RNA-seq, Chen et al. (9) found that infection of A549 cells for 8 h with A. fumigatus strain B5233 strongly induced the expression of genes encoding cytokines like IL-6, IL-8, and TNF-α. They also found that A. fumigatus induced the expression of *ARC* (activity-regulated cytoskeleton-associated protein) and several *EGR* (early growth response) genes. Microarray studies of A549 cells infected with A. fumigatus Af293 performed by Sharon et al. (11) yielded similar results. We found that A. fumigatus strains Af293 and CEA10 induced a broader response after 16 h of infection. Both previous studies found that the host pathways enriched in response to A. fumigatus included cytokine-cytokine receptor interaction and MAPK signaling pathways. Our more detailed pathway analysis identified multiple cytokine signaling pathways that were predicted to be induced by A. fumigatus infection, including the PDGF BB, IL-1α, IL-1β, IL-2, and IL-17A pathways. Also, the p38 MAPK, MAP kinase kinase 1 and 2 (MAP2K1/2), and ERK pathways were predicted to be upregulated in response to A. fumigatus. Although only Chen et al. (9) found that the JAK-STAT pathway was induced by A. fumigatus, we identified multiple other proinflammatory pathways that were predicted to be activated, including the NFKB, RelA, TLR2, TLR4, and TLR7 pathways. A key difference between the previous studies and the one reported here is that both previous studies used an 8-h time point, whereas we used 6- and 16-h time points. It is probable that the longer infection time used in the current experiments induced a stronger and broader response in A549 cells.

A notable finding was that although both strains of A. fumigatus germinated similarly on A549 cells, strain CEA10 induced a much stronger transcriptional response at 6 h than did strain Af293. After 16 h of infection, both strains induced a broad transcriptional response, but CEA10 altered the expression of more host cell genes, especially those that were downregulated. It has been found that strain CEA10 induces greater lethality than strain Af293 after intranasal or intratracheal inoculation in immunocompetent mice (20, 54). The enhanced virulence of strain CEA10 has been attributed to more rapid germination. However, our transcriptional results suggest that strain CEA10 has a greater effect on host cells than strain Af293 independently of germination rate.

Although strains CEA10 and Af293 induced somewhat different transcriptional responses, they were predicted to activate most of the same host signaling pathways after 16 h of infection. Among these pathways were the PDGF BB and PGR pathways. Inhibiting PDGFR and PGR reduced the endocytosis of both strains of A. fumigatus in an additive manner, suggesting that PDGF BB and PGR may function in different pathways to induce endocytosis. Previously, we found that PDGF BB was required for the maximal endocytosis of C. albicans by endothelial and oral epithelial cells (16). PDGFR was also required for maximal damage of endothelial cells by R. delemar (14). Collectively, these data suggest that the PDGF BB signaling pathway mediates the interactions of multiple fungi with normally nonphagocytic host cells (14, 16).

Our finding that the PGR inhibitor mifepristone inhibited the endocytosis of both strains of A. fumigatus by A549 cells indicates that progesterone signaling induces the endocytosis of this fungus, thus supporting the upstream regulator analysis. The PGR pathway is also predicted to be activated in A549 cells exposed to R. delemar and R. oryzae (14), but not in endothelial cells or oral epithelial cells infected with C. albicans (16). While the PGR pathway was not predicted to be activated in A549 cells infected with influenza B virus or P. aeruginosa (52, 53), these studies did not evaluate sufficient time points to be able to draw conclusions about whether the PGR pathway is activated by nonmold pathogens.

There are limitations to our study. First, monolayers of A549 cells do not capture the cellular diversity of the airway epithelium. The lack of cellular diversity likely results in the absence of some specialized signaling networks present *in vivo* that play important roles in epithelial function, including the response to inhaled microbes. Second, the initial interaction between the conidia and host cells is also not perfectly recapitulated because the A549 cells are a cancer cell line that are grown submerged in liquid medium, while lung epithelial cells *in vivo* exist at an air-liquid interface. Third, the interpretation of our pathway analysis is not straightforward due to interconnectedness of many host signaling pathways. Our upstream regulator analysis draws from a curated database of molecular and genetic interactions in a wide variety of experimental contexts, many of which are not relevant to the interaction between fungi and epithelial cells. The significant overlap in regulated gene sets among regulators can lead to misattribution of a potential pathway regulation. Therefore, it is important to verify the results of the upstream regulator analysis with an orthogonal approach to determine if a given pathway is modulated during fungal infection or involved in disease establishment or progression. Using inhibitors of PDGF BB and PGR, we were able to verify that these pathways were indeed involved in the response of A549 cells to A. fumigatus.

In summary, our dual-species transcriptomics approach combined with the Upstream Regulator Analytic identified multiple signaling pathways in pulmonary epithelial cells that were predicted to be activated by infection with two different clinical isolates of A. fumigatus. Among these pathways, the PDGF BB and PGR pathways were predicted to be activated by fungal infection, and experiments with small-molecule inhibitors indicated that these pathways governed the endocytosis of A. fumigatus in an additive manner. We also identified a set of A. fumigatus genes whose expression was correlated, either directly or indirectly, with the host transcriptional response to infection. Thus, these data sets will constitute a valuable resource for further studies to understand the interaction of A. fumigatus and perhaps other pulmonary pathogens with host cells during infection.

## MATERIALS AND METHODS

### *In vitro* A. fumigatus infections.

Infections were performed as previously described (12). Briefly, A. fumigatus strains Af293 and CEA10 were grown on Sabouraud agar (Difco, Detroit, MI) at 37°C for 5 to 7 days prior to use. Conidia were harvested by rinsing the plate with phosphate-buffered saline (PBS) containing 0.1% Tween 80 (Sigma-Aldrich), followed by counting using a hemacytometer. The A549 type II pneumocyte cell line (American Type Culture Collection) was grown in F-12 K medium (ATCC) containing 10% fetal bovine serum (Gemini Bio-Products) and 1% streptomycin and penicillin (Irvine Scientific) in 5% CO_2_ at 37°C. One day prior to infection, 7 × 10^5^ A549 cells were added to each well of a 6-well tissue culture plate. The next day, the A549 cells were rinsed with serum-free F-12 K medium. At this point, 1 × 10^6^ conidia of each strain were added to individual wells of the 6-well tissue culture plate. Control wells contained uninfected A549 cells. After 6 and 16 h of incubation, total RNA was extracted and then purified using the RiboPure kit (Ambion, Life Technology) and the RNA Clean & Concentrator kit (Zymo Research), respectively.

### RNA-seq data generation and analysis.

All RNA-seq libraries (non-strand specific, paired end) were prepared with the TruSeq RNA sample prep kit (Illumina). The total RNA samples were subjected to poly(A) enrichment as part of the TruSeq protocol. One hundred nucleotides (nt) of sequence was determined from both ends of each cDNA fragment using the HiSeq platform (Illumina) according to the manufacturer’s protocol. Sequencing reads were annotated and aligned to the human reference genome (Ensembl GRCh38) using TopHat2 (55). The alignment files from TopHat2 were used to generate read counts for each gene, and differential expression between specific experimental groups was determined using the EdgeR package from Bioconductor (56). A gene was considered differentially expressed if the false discovery rate (FDR) for differential expression was less than 0.01 and the absolute log_2_-fold change was greater than or equal to one. We used the Upstream Regulator Analytic of IPA (Ingenuity Systems) to identify signaling proteins that were potentially activated or repressed during the course of infection. This analysis determines the overlap between lists of differentially expressed genes and an extensively curated database of regulator-target gene relationships. It then considers the direction of the gene expression changes to make predictions about activation or repression of specific pathways. Descriptions and annotations of the A. fumigatus genes were obtained from FungiDB (https://fungidb.org/fungidb/app) using the default setting (57).

### Invasion assay.

The endocytosis of A. fumigatus by A549 cells was determined using our differential fluorescence assay (6, 58). Af293 and CEA10 strains constitutively expressing green fluorescent protein (GFP) were used in this assay (6). Briefly, 2 × 10^5^ A549 cells were cultured on circular glass coverslips coated with fibronectin in a 24-well tissue culture plate. The next day, the confluent A549 cells were rinsed twice with F-12 K medium and then infected with 10^5^ germlings of the GFP-expressing A. fumigatus strains. For studies with inhibitors, the A549 cells were incubated with 10 μM mifepristone Ru-486 (M8046; Sigma) and/or 10 μM PDGFR tyrosine kinase inhibitor III (SC-204173; Santa Cruz Biotechnology) in 0.1% dimethyl sulfoxide (DMSO) for 45 min before infection. Control cells were incubated in medium containing 0.1% DMSO alone. After 2.5 h of incubation, the cells were rinsed with 1 mL Hanks balanced salt solution (HBSS) and fixed with 3% paraformaldehyde. The noninternalized organisms were stained with a rabbit anti-A. fumigatus antibody (Meridian Life Science, Inc.), followed by an Alexa Fluor 568-labeled secondary antibody (Life Technologies). The coverslips were mounted inverted, and the invasion was observed and at least 100 organisms per coverslip counted. Each experiment was performed three times in triplicate.

### Data availability.

All of the raw sequencing reads from this study are available at the NCBI Sequence Read Archive (SRA) under BioProject accession number PRJNA399754. The specific sample accession numbers are presented in Table S1.
